# Joint‐specific measures improve risk adjustment in total knee arthroplasty: A machine learning approach

**DOI:** 10.1002/ksa.70480

**Published:** 2026-06-15

**Authors:** Dirk Müller, Amna Gillani, Michael T. Hirschmann, Igor Lazic, Florian Hinterwimmer, Anabel Arber, Florian Krüger, Georg Matziolis, Rüdiger von Eisenhart‐Rothe

**Affiliations:** ^1^ Department of Orthopaedic Surgery, TUM Klinikum Rechts der Isar, TUM School of Medicine and Health Technical University of Munich Munich Germany; ^2^ Institute for AI and Informatics in Medicine, TUM Klinikum Rechts der Isar, TUM School of Medicine and Health Technical University of Munich Munich Germany; ^3^ Department of Orthopaedic Surgery and Traumatology Kantonsspital Baselland Bruderholz Switzerland; ^4^ Department of Orthopaedic Surgery, Waldkliniken Eisenberg, Jena University Hospital Friedrich Schiller University Jena Eisenberg Germany; ^5^ AE—German Society for Endoprosthetics Freiburg Germany

**Keywords:** complication, feature importance, machine learning, risk adjustment, total knee arthroplasty (TKA)

## Abstract

**Purpose:**

Accurate risk adjustment in total knee arthroplasty (TKA) is essential for outcome prediction and quality assessment. Most existing prediction models rely solely on patient demographics and comorbidities and do not account for joint‐specific pathology. This study evaluated whether incorporating radiographic and clinical joint‐specific parameters improves machine learning (ML)–based risk adjustment and prediction of postoperative complications and residual pain following TKA.

**Methods:**

A retrospective analysis was performed on 1207 primary TKA procedures conducted at a single academic centre between 2018 and 2022. Three outcomes at 1 year were analysed: residual pain (Visual Analogue Scale [VAS] ≥ 4), any complications and major complications. Predictor variables included patient‐related factors (e.g., age, body mass index, American Society of Anesthesiologists score, comorbidities) and joint‐specific parameters (e.g., limb alignment, range of motion) derived from preoperative radiographs and clinical assessment. Binary classification models were developed using a stacked gradient‐boosting ensemble combining XGBoost and CatBoost. For each outcome, models using patient‐specific variables alone were compared with models incorporating both patient‐ and joint‐specific variables. Model performance was evaluated using accuracy, sensitivity, specificity and area under the receiver operating characteristic curve (AUC).

**Results:**

Incorporating joint‐specific parameters significantly improved prediction of complications. For major complications, the combined model achieved an AUC of 0.74 compared with 0.66 using patient variables alone. For any complications, the AUC increased from 0.64 to 0.72. No improvement was observed for predicting residual pain. The most influential joint‐specific predictors included prior septic surgery, large bone defects, Kellgren–Lawrence Grade < 3, prior ligament reconstruction and preoperative knee flexion < 70°.

**Conclusion:**

Inclusion of joint‐specific features improved ML‐based prediction of postoperative complications following TKA, but did not improve prediction of residual pain. These findings suggest that joint‐specific parameters may enhance risk adjustment for postoperative complications in TKA.

**Level of Evidence:**

Level III retrospective cohort study.

**Abbreviations**
ASA: American Society of Anesthesiologists
AUC: area under the curve
BMI: body mass index
CMS: Centers for Medicare and Medicaid Services
K&L: Kellgren–Lawrence grade
ML: machine learning
NHS: National Health Service (United Kingdom healthcare system)
PROMs: patient‐reported outcome measures
ROC: receiver operating characteristic
SHAP: SHapley Additive exPlanations
SMOTE: Synthetic Minority Oversampling Technique
THA: total hip arthroplasty
TKA: total knee arthroplasty
VAS: Visual Analogue Scale (for pain)
XGBoost: eXtreme Gradient Boosting (a regularizing gradient boosting ML framework)

## INTRODUCTION

Total knee arthroplasty (TKA) is a widely performed and generally successful surgical intervention aimed at relieving pain and restoring function in patients with advanced knee osteoarthritis. Despite its effectiveness, postoperative complications—ranging from wound issues to infections and implant failures—remain a significant concern, with reported rates varying between 1% and 6% [4, 5, 35, 51], depending on patient characteristics and surgical factors. Identifying patients at elevated risk for such complications is essential for improving outcomes, guiding perioperative management and informing shared decision‐making.

Traditional risk assessment models for TKA have primarily relied on patient‐related factors such as age, gender, body mass index (BMI) and comorbidities including diabetes, cardiovascular disease and smoking status [3, 4, 5, 9, 15, 39, 40, 45, 48, 51]. Although these models provide useful population‐level insights, they often lack precision for individualized risk prediction.

In addition to general patient‐related risk factors, local joint conditions may play a critical role in determining postoperative outcomes following TKA. Structural abnormalities such as severe deformity, ligament instability, reduced range of motion or bone defects can increase surgical complexity, prolong operative time and affect soft tissue balancing. These factors may contribute to complications such as instability, stiffness or infection [10, 30, 41]. From a biomechanical perspective, altered limb alignment and joint kinematics influence load distribution and implant positioning, which may further affect postoperative outcomes.

Recent advances in machine learning (ML) have shown promise in addressing this limitation [13, 16, 17, 18, 20, 22, 26, 29, 32, 33, 37, 42]. ML algorithms can analyse complex high‐dimensional data to detect non‐linear relationships and interactions among variables, offering the potential for more accurate and personalized risk prediction [23]. Prior ML models in TKA have demonstrated varying performance in predicting complications, readmissions and patient‐reported outcomes, with reported areas under the curve (AUCs) ranging from 0.60 to 0.87. However, the predictive value of these models has often been limited by a narrow focus on patient demographics and comorbidities.

To our knowledge, no prior study has incorporated joint‐specific clinical and radiographic features—such as range of motion, coronal and sagittal alignment or the presence of structural abnormalities—into ML‐based risk prediction models for TKA. These joint‐specific parameters, routinely assessed in clinical practice and preoperative imaging, may offer valuable information not captured by general health metrics alone.

The aim of this study was to evaluate whether joint‐specific clinical and radiographic features improve ML‐based risk adjustment following TKA. We hypothesized that integrating these joint‐specific features into a stacked gradient‐boosting ensemble model would improve prediction of postoperative complications and residual pain compared with patient‐specific variables alone.

## MATERIALS AND METHODS

### Data source

We retrospectively analysed all primary TKAs performed at our university hospital between January 2018 and December 2022 (*n* = 1423). According to our institutional protocol, all patients underwent standardized clinical evaluations preoperatively, at 6 weeks postoperatively and at 1 year postoperatively. Informed consent was obtained from all individual participants included in the study. Ethical approval was granted by the local ethics committee of the Technical University of Munich (IRB approval number 714/20 S).

### Data screening, cleaning and preparation

**Figure 1:**
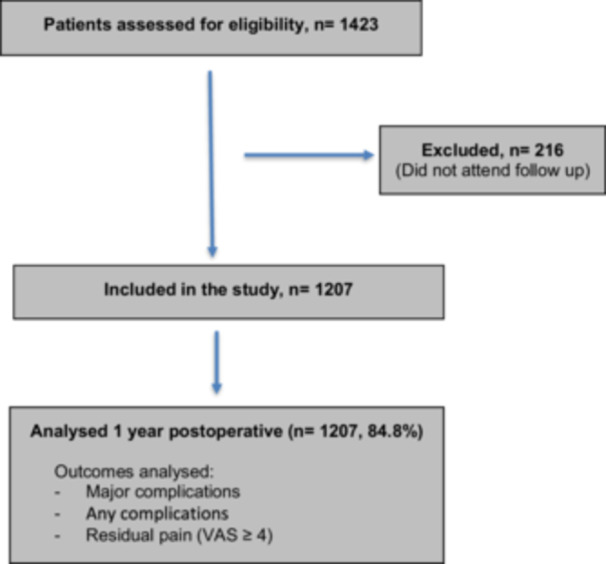
STROBE flow diagram of patient selection, follow‐up and inclusion in the analysis. STROBE, Strengthening the Reporting of Observational Studies in Epidemiology; VAS, Visual Analogue Scale.

Data were missing for 216 patients who did not attend the 6‐week and 1‐year follow‐up examinations, and these patients were excluded from the analysis. The final study cohort comprised 1207 patients, corresponding to a follow‐up rate of 84.8%. The Strengthening the Reporting of Observational Studies in Epidemiology (STROBE) flow diagram of patient inclusion and follow‐up is shown in Figure 1.

A complete clinical examination, including measures of range of motion, was performed before surgery, 6 weeks postoperative and 1 year postoperative. The radiographic evaluation included a standing long leg radiograph, a lateral radiograph of the knee and a skyline view of the patella. Joint‐specific radiographic measurements were performed manually by orthopaedic residents under the supervision of orthopaedic and radiology consultants. Patient characteristics are displayed in Table 1, joint‐specific measures are displayed in Table 2.

**Table 1:** Patient characteristics of the study cohort.

Patient characteristics	(*n* = 1207)
Gender female/male, *n* (%)	681 (56.4%)/526 (43.6%)
Age, years, median (range)	69 (21−98)
BMI, kg/m^2^, median (range)	28.3 (14.9−60.5)
ASA = 1, *n* (%)	102 (8.5%)
ASA = 2, *n* (%)	809 (67.0%)
ASA = 3, *n* (%)	290 (24.0%)
ASA = 4, *n* (%)	6 (0.5%)
GFR, mL/min, median (range)	82 (4−128)

*Note*: Categorical variables are presented as absolute numbers and percentages; continuous variables are presented as median and range.

Abbreviations: ASA, American Society of Anesthesiologists; BMI, body mass index; GFR, glomerular filtration rate.

**Table 2:** Joint‐specific radiographic and clinical measures.

Joint‐specific measures	(*n* = 1207)
Kellgren–Lawrence [31] Grade 2, *n* (%)	235 (19.5%)
Kellgren–Lawrence Grade 3, *n* (%)	458 (37.9%)
Kellgren–Lawrence Grade 4, *n* (%)	514 (42.6%)
Varus knees, *n* (%)	871 (72.2%)
Hip knee angle in varus knees, °, median (range)	6.2° (0.1°–24.7°)
Valgus knees, *n* (%)	336 (27.8%)
Hip knee angle in valgus knees, °, median (range)	5.8° (0.1°–30.3°)
Medial proximal tibial angle (MPTA), °, median (range)	87.7° (71.0°−102.9°)
Joint line conversion angle (JLCA), °, median (range)	4.0° (0.1°−19.6°)
Lateral distal femur angle (LDFA), °, median (range)	88.1° (73.6°−99.0°)
Tibial slope (Dejour and Bonnin [12]), °, median (range)	9.3° (−8.1° to 30.4°)
Extension deficit, °, median (range)	0.0° (0.0°−30.0°)
Flexion, °, median (range)	120.0° (30.0°−140.0°)
Range of motion, °, median (range)	110° (20°−140°)

*Note*: Categorical variables are presented as absolute numbers and percentages; continuous variables are presented as median and range.

### Risk factors

Risk factors were categorized into two groups: patient‐specific and joint‐specific. Patient‐specific risk factors were based on the criteria established by the American Association of Hip and Knee Surgeons (AAHKS) for risk stratification in arthroplasty care [1, 4].

Joint‐specific risk factors were identified through a three‐stage Delphi process involving 14 experienced arthroplasty surgeons [44]. The selection of these variables was guided by clinical relevance and existing evidence suggesting an association with surgical complexity, implant positioning, soft tissue balancing and postoperative function. The aim was to include parameters that reflect local joint pathology and biomechanical conditions, which are not captured by general patient‐related variables (Table 3).

**Table 3:** Patient‐specific and joint‐specific risk factors for complications following total knee arthroplasty.

Patient‐specific risk factors	*n* (%)	Joint‐specific risk factors (Delphi consensus)	*n* (%)
Male gender	526 (43.6%)	Prior septic surgery	34 (2.8%)
Age < 55 years	134 (11.1%)	Large bone defect	226 (18.7%)
Age > 80 years	158 (13.1%)	Implanted foreign material	66 (5.5%)
Skin lesions	106 (8.8%)	Valgus > 10°	86 (7.1%)
GFR < 30 mL/min	20 (1.7%)	Varus > 15°	50 (4.1%)
Peripheral artery disease	12 (1.0%)	Extension deficit > 10°	81 (6.7%)
Nickel allergy	95 (7.9%)	Flexion < 70°	21 (1.7%)
Chronic pain patient	135 (11.2%)	Kellgren–Lawrence Grade < 3	235 (19.5%)
ASA ≥ 3	296 (24.5%)	Patella baja (Caton–Deschamps index < 0.6) [8]	89 (7.4%)
BMI > 40 kg/m^2^	68 (5.6%)	Prior bone surgery	179 (14.8%)
Chronic anticoagulant use	343 (28.4%)	Genu recurvatum > 10°	16 (1.3%)
Rheumatoid arthritis	46 (3.8%)	Prior ligament reconstruction	90 (7.5%)
Diabetes	165 (13.7%)		
Smoking	142 (11.8%)		
Depression/psychiatric disease	114 (9.4%)		

Abbreviations: ASA, American Society of Anesthesiologists; BMI, body mass index; GFR, glomerular filtration rate.

### Outcome labels

The primary outcome was the occurrence of postoperative complications within the first year following TKA. Complications were defined as ‘any deviation from the normal postoperative course’, in accordance with the definition by Dindo et al. [14], and classified based on the standardised list of 22 complications proposed by the Knee Society [21, 25]. Complications were further categorised as major or minor. Major complications were defined as events requiring revision surgery or resulting in death related to the index procedure and corresponded to Grades 3–5 of the Clavien–Dindo classification [14], as modified for TKA by Iorio et al. [25].

The secondary outcome was residual pain 1 year after TKA, defined as a Visual Analogue Scale (VAS) pain score ≥ 4, a threshold commonly used to indicate moderate to severe chronic musculoskeletal pain [6]. Based on these definitions, three binary outcome labels were used for the ML models: major complication, any complication (major or minor) and residual pain (Table 4). Due to the retrospective nature of the study, no blinding of outcome or predictor assessment was performed.

**Table 4:** Frequency and classification of postoperative complications in the patient cohort.

	Absolute (*n*)	Relative (%)
Major complication (revision surgery)	55	4.6%
Periprosthetic joint infection	15	1.2%
Arthrofibrosis	13	1.1%
Hemarthrosis	7	0.6%
Instability	5	0.4%
Wound healing complication	3	0.2%
Aseptic loosening	3	0.2%
Retropatellar arthrosis (with revision surgery)	3	0.2%
Periprosthetic fracture	2	0.2%
Patellar tendon rupture	2	0.2%
Death related to surgery	2	0.2%
Minor complication (no revision surgery)	50	4.1%
Wound healing complication	17	1.4%
Arthrofibrosis	7	0.6%
Partial rupture of the quadriceps tendon	5	0.4%
Erysipel	5	0.4%
Hemarthrosis	4	0.3%
Deep vein thrombosis	4	0.3%
Pulmonary embolism	3	0.2%
Renal failure	2	0.2%
Partial rupture of the patellar tendon	1	0.1%
Patellar fracture	1	0.1%
Drop foot	1	0.1%

### Descriptive statistics

Descriptive statistical analyses were performed using IBM SPSS Statistics for Windows, Version 29.0 (IBM Corp.). The normality of continuous variables was assessed using the Shapiro–Wilk test. Variables with a normal distribution are reported as mean ± standard deviation, while non‐normally distributed variables are presented as median with range.

### ML model

We developed a supervised ML pipeline to perform binary classification for the three target variables using an ensemble of Extreme Gradient Boosting (XGBoost) and CatBoost models. XGBoost is a gradient boosting ML method that builds a series of decision trees, where each new tree focuses on correcting the errors made by the previous ones. Through this stepwise learning process, the model progressively improves its predictions. CatBoost is another gradient boosting algorithm that works in a similar way but is particularly well suited for handling categorical variables. This is relevant because many of our predictors are categorical clinical variables (e.g., gender, comorbidities and prior surgeries).

For each outcome, we trained two models: one using only patient‐specific features and another using both patient‐specific and joint‐specific features. Multiple XGBoost and CatBoost learners with different hyperparameter configurations were trained and combined in a stacked ensemble. At the last stage, predictions from individual models were combined to make the final decision.

The data were divided into five equal parts. In each iteration, the model was trained on four parts and tested on the remaining part. This process was repeated five times so that each part served once as the test set. Within each training fold, we applied the Synthetic Minority Oversampling Technique (SMOTE) to address class imbalance. Model hyperparameters were tuned using random search within the training folds.

We assessed model performance using the area under the receiver operating characteristic curve (AUC), accuracy, sensitivity and specificity. To ensure clinical relevance and comparability across models, classification thresholds were determined by fixing sensitivity at 0.6 for all models, and corresponding performance metrics were calculated at this operating point. Differences in AUC between models were evaluated using the DeLong test for correlated receiver operating characteristic (ROC) curves, with statistical significance defined as a two‐sided *p* value < 0.05.

We used SHapley Additive exPlanations (SHAP) values to indicate how much each feature contributes to the model's prediction. We report absolute SHAP values for the model with the greatest contribution within the ensemble.

## RESULTS

**Figure 2:**
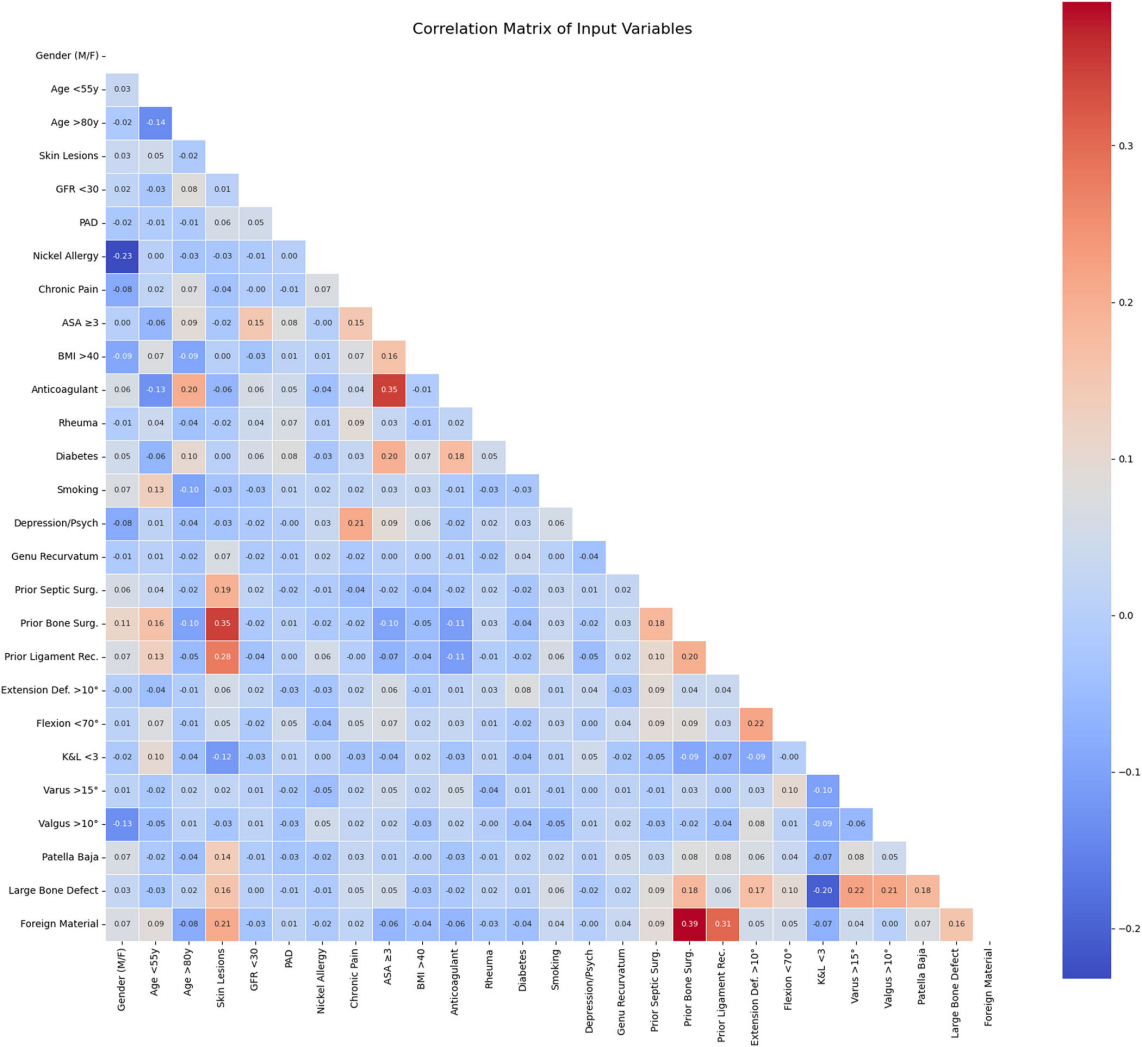
Heatmap of the correlation matrix illustrating pairwise correlations among all variables included in the analysis, with colour intensity indicating the strength and direction of the correlations. ASA, American Society of Anesthesiologists; BMI, body mass index; GFR, glomerular filtration rate; K&L, Kellgren–Lawrence grade; PAD, peripheral arterial disease.

The correlation matrix of all input variables is presented in Figure 2.

A summary of the ML model results for the three outcome labels—residual pain, any complication and major complication—is presented in Table 5. Incorporating joint‐specific features improved both accuracy and AUC for predicting complications.

**Table 5:** Summary of machine learning model performance for the three outcome labels: residual pain, any complication and major complication.

	Residual pain			Any complication in 1 year			Major complication in 1 year		
	Patient‐specific features	Patient‐ and joint‐specific features	DeLong *p* value	Patient‐specific features	Patient‐ and joint‐specific features	DeLong *p* value	Patient‐specific features	Patient‐ and joint‐specific features	DeLong *p* value
Accuracy	61%	61%		58%	69%		65%	72%	
Sensitivity	60%	60%		60%	60%		60%	60%	
Specificity	61%	61%		58%	70%		66%	73%	
AUC (95% CI)	0.63 (0.59–0.66)	0.63 (0.59–0.66)	0.961	0.64 (0.59–0.69)	0.72 (0.67–0.77)	0.007	0.66 (0.58–0.73)	0.74 (0.68–0.80)	0.019

Abbreviations: AUC, area under the curve; CI, confidence interval.

### Outcome label: Major complication

**Figure 3:**
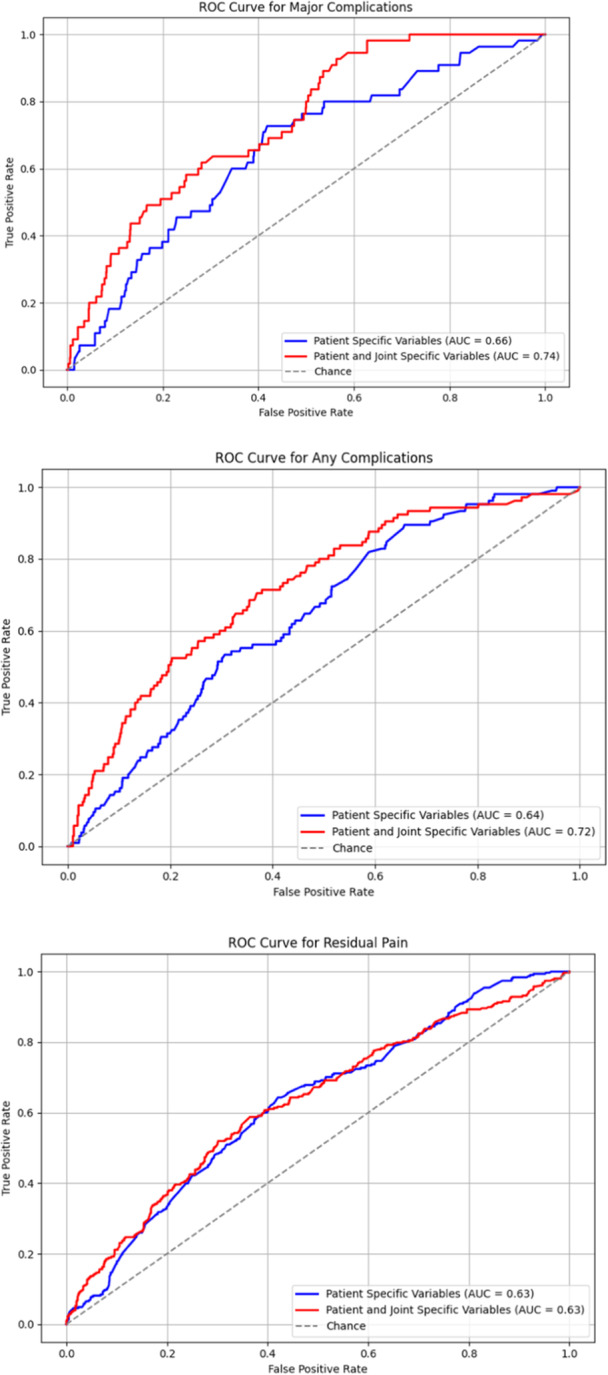
Receiver operating characteristic (ROC) curves for predicting major complications, any complications and residual pain (VAS ≥ 4), comparing models with and without joint‐specific features. Model performance is expressed as the area under the curve (AUC). VAS, Visual Analogue Scale.

**Figure 4:**
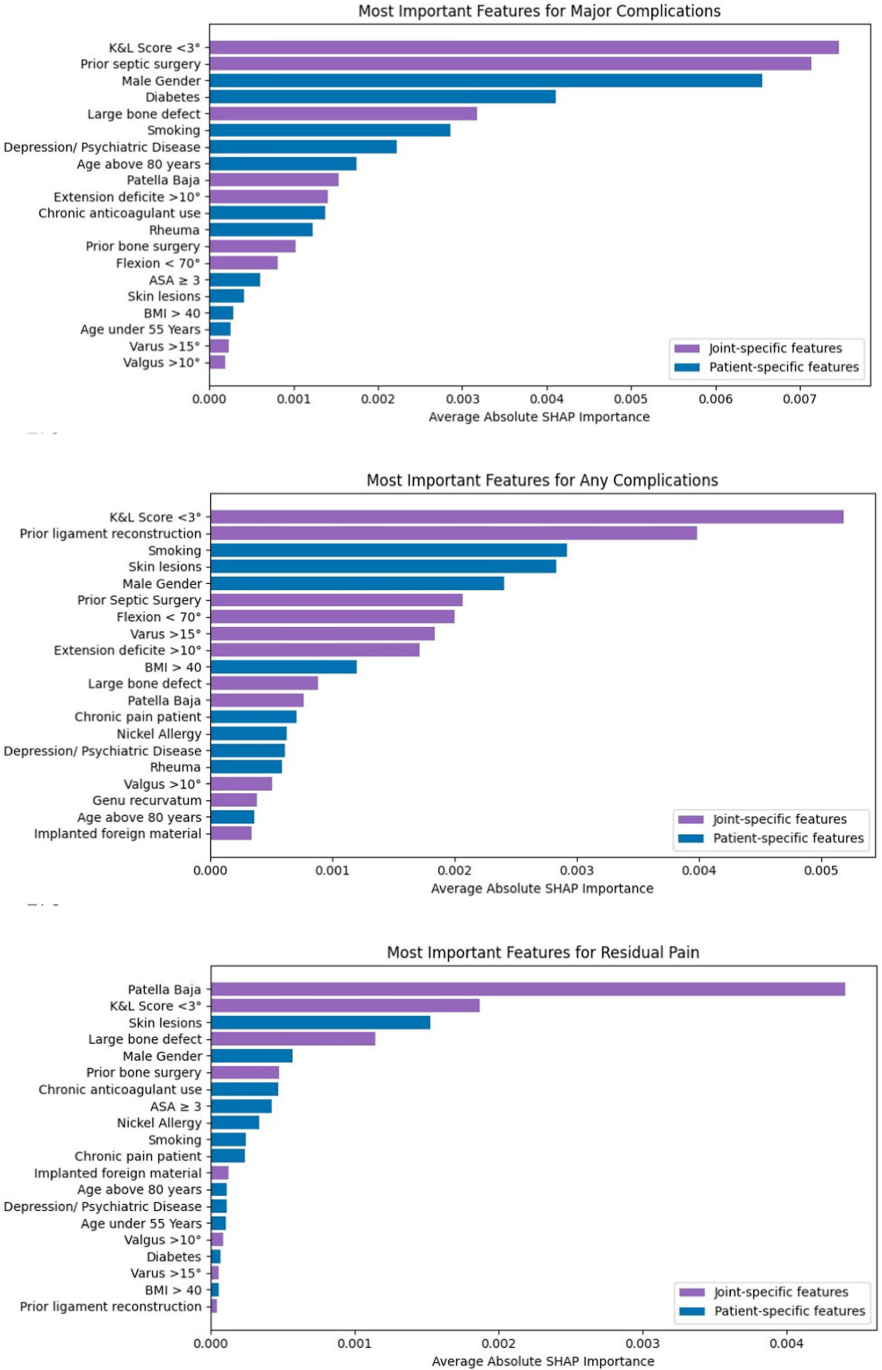
Top 20 predictive features for the outcomes major complications, any complications and residual pain (VAS ≥ 4). Feature importance for each outcome was calculated using SHAP values from the ensemble model. ASA, American Society of Anesthesiologists; BMI, body mass index; K&L, Kellgren–Lawrence grade; SHAP, SHapley Additive exPlanations.

The ROC for the outcome label ‘major complication’ is shown in Figure 3. Incorporating joint‐specific features significantly increased the AUC from 0.66 to 0.74 (*p* = 0.019), indicating improved predictive performance. The most influential features for predicting major complications were the joint‐specific features Kellgren–Lawrence Grade < 3, followed by prior septic surgery. The most important patient‐specific variables were male gender and diabetes, as shown in Figure 4.

### Outcome label: Any complication

The addition of joint‐specific features significantly increased the AUC from 0.64 to 0.72 (*p* = 0.007), demonstrating enhanced model performance. The top joint‐specific feature for any complication was also Kellgren–Lawrence Grade < 3, followed by prior ligament reconstruction. The most important patient‐specific features were smoking and skin lesions.

### Outcome label: Residual pain (VAS ≥ 4)

The ML model demonstrated limited performance in predicting residual pain using only patient‐specific features, with an AUC of 0.63. The inclusion of joint‐specific features did not improve the prediction of residual pain. The most influential predictor of residual pain following TKA was patella baja prior to TKA.

## DISCUSSION

The key finding of this study is that incorporating joint‐specific radiographic and clinical measures improved risk adjustment in TKA when using ML. Specifically, the ensemble model demonstrated enhanced predictive performance for postoperative complications with the inclusion of joint‐specific features. To our knowledge, this is the first study to specifically evaluate the incremental value of detailed joint‐specific clinical and radiographic parameters in a risk adjustment model for TKA.

Previous studies evaluating ML models to predict TKA complications have shown variable performance. Harris et al. used a Least Absolute Shrinkage and Selection Operator (LASSO)‐based model to predict 30‐day complications after TKA and total hip arthroplasty (THA), reporting an AUC of 0.64 for any complication [20]. Aram et al., analysing data from a national joint registry, reported an AUC of 0.705 using a random forest model to predict implant survivorship [2]. Mohammadi et al. achieved an AUC of 0.86 for predicting 30‐day readmissions using a neural network [42]. In contrast, El Galaly et al., using data from the Danish Knee Arthroplasty Registry, found that multiple ML approaches failed to develop a clinically useful model for predicting early revisions within 2 years, despite including known presurgical risk factors [16].

Various studies have been published on ML models predicting patient satisfaction and patient‐reported outcome measures (PROMs) after TKA, reporting AUCs between 0.60 and 0.87 [17, 18, 19, 24, 34, 46]. In particular, Huber et al. used an XGBoost model on National Health Service (NHS) data and achieved an AUC of 0.87 for predicting postoperative pain (VAS) [24]. However, our model showed limited predictive performance for residual pain, with no improvements from joint‐specific parameters.

The AAHKS has proposed a set of nine risk factors to enhance existing TKA risk‐adjustment models [1]. In a previous study, we demonstrated that an XGBoost‐based ML model incorporating AAHKS‐defined risk factors achieved moderate discriminatory performance for predicting major postoperative complications following TKA (AUC = 0.68) [43]. In contrast, the present study demonstrates that a broader integration of joint‐specific variables and imaging‐derived parameters into the risk‐adjustment framework resulted in improved model performance, with an AUC of 0.74.

In a collaborative study between the FORCE‐TJR registry and AAHKS, combining clinical variables with Centers for Medicare and Medicaid Services (CMS) administrative data improved risk prediction. Specifically, adding five clinical variables (contralateral arthritis severity, BMI, preoperative 36‐Item Short Form Health Survey (SF‐36) physical function score, Charlson comorbidity index and smoking status) increased the AUC from 0.65 (administrative data only) to 0.79 using logistic regression [4].

In our study, patient‐specific features with high importance were smoking, skin lesions, diabetes and history of psychiatric disease—consistent with established risk factors in the literature [1, 3, 4, 5, 7, 9, 39, 45, 47, 50]. Gender also showed a notable trend, with males demonstrating a higher predicted risk of complications. This finding is nuanced in the literature; while Boyer et al. reported higher rates of septic revisions in males [7], other studies, such as that by Schiffner et al., found no association between gender and implant survival [50]. Chronic anticoagulant use emerged as a risk factor for major complications, in line with previous work by Johnson et al., which demonstrated increased risks of revision and wound disruption among anticoagulated patients [27].

Among the 12 joint‐specific features identified in our Delphi study [44], the most predictive for complications included Kellgren–Lawrence Grade < 3, prior septic surgery, prior ligament reconstruction, large bone defects and knee flexion < 70° prior to TKA. Patella baja was not predictive of complications, although patella baja was strongly associated with residual pain. Lower preoperative Kellgren–Lawrence grades are known to be associated with residual pain and dissatisfaction following TKA [11]. Interestingly, we also observed an association between low preoperative Kellgren–Lawrence grades and complications in our cohort.

These findings are consistent with previous evidence indicating that several joint‐specific parameters are associated with postoperative outcomes. Limited preoperative range of motion has been linked to postoperative stiffness [49], while lower radiographic osteoarthritis grades have been associated with persistent postoperative pain and dissatisfaction [11, 38]. Thus, joint‐specific features may capture clinically relevant information that is not reflected by systemic patient characteristics alone.

The overall rate of major complications in our cohort (4.6%) lies at the upper end of the range reported in the existing literature (1%−6%) [4, 5, 35, 51]. This finding should be interpreted in the context of our institution being a tertiary academic referral centre, which receives a substantial number of complex cases from smaller hospitals. Patients with severe deformities or significant comorbidities are frequently referred because operative treatment is considered to carry an increased risk of complications at the referring institutions. This referral bias is likely to have contributed to the comparatively high complication rate observed in the present series. Furthermore, minor complications were systematically documented. Several of these events, including minor wound‐healing disturbances that did not require additional intervention, are not consistently reported in comparable studies. This difference in reporting practices may have further influenced the overall complication rate reported in this study.

Comparisons with previous studies are also complicated by heterogeneous outcome definitions. Many U.S.‐based models focus on 30‐day readmissions, given the availability of CMS data [4], while other studies use revision rates [16]. For ML models predicting patient satisfaction, different studies have employed varying PROMs, which limits the comparability of both methodologies and results across studies [28].

### Limitations

This study has several limitations. First, ML models identify associations that do not imply causality. Feature importance reflects the contribution of variables to model predictions but does not establish a direct causal relationship with outcomes. Therefore, the findings should be interpreted as hypothesis‐generating rather than confirmatory, and the improved predictive performance associated with joint‐specific variables does not necessarily indicate a causal relationship with postoperative complications or residual pain. Second, no external validation was performed, and the generalizability of the model to independent cohorts remains uncertain. Third, the relatively small sample size and limited number of complication events increase the risk of overfitting [36]. This is further exacerbated by the inherent class imbalance in arthroplasty datasets, where low complication rates may lead to misleadingly high accuracy while failing to detect clinically relevant events [22]. To mitigate this, model performance was evaluated at a predefined sensitivity of 0.6 rather than relying solely on accuracy.

## CONCLUSION

Incorporating joint‐specific radiographic and clinical features improved ML‐based prediction of postoperative complications following TKA, but did not improve prediction of residual pain. These findings suggest that joint‐specific parameters may enhance risk adjustment for postoperative complications in TKA. Future studies using larger, prospective datasets with external validation are needed to confirm the generalizability and clinical applicability of these findings.

## AUTHOR CONTRIBUTIONS

**Dirk Müller**: Conceptualization; methodology; data cleaning; formal analysis; investigation; visualization; writing—original draft. **Amna Gillani**: Methodology; software; formal analysis; visualization; writing—review and editing. **Michael T. Hirschmann**: Writing—review and editing; supervision. **Igor Lazic**: Conceptualization; methodology; writing—review and editing. **Florian Hinterwimmer**: Methodology; software; writing—review and editing. **Anabel Arber**: Data collection; investigation. **Florian Krüger**: Data collection; investigation. **Georg Matziolis**: Writing—review and editing. **Rüdiger von Eisenhart‐Rothe**: Conceptualization; methodology; supervision; writing—review and editing. All authors have read and approved the final version of the manuscript.

## CONFLICT OF INTEREST STATEMENT

The authors declare no conflicts of interest.

## ETHICS STATEMENT

Ethical approval was granted by the local Ethics Committee of the Technical University of Munich (IRB approval number 714/20 S). Informed consent was obtained from all patients prior to study inclusion.

## Supporting information

Supplementary Material ‐ Inter‐rater reliability.

Supplementary Material ‐ Tripod‐Checklist.

## Data Availability

The datasets generated and/or analysed during the current study are available from the corresponding author on reasonable request.
