# Supplementary material for: Joint‐specific measures improve risk adjustment in total knee arthroplasty: A machine learning approach
Source: Knee Surg Sports Traumatol Arthrosc. 2026 Jun 15;34(7):2661–72. doi: 10.1002/ksa.70480 (PMC13327454; doi:10.1002/ksa.70480)
Supplement: Supplementary file 1 — Supplementary Material ‐ Inter‐rater reliability. [file KSA-34-2661-s001.pdf]

Supplementary Material – Inter-rater reliability of radiologic measurements

Inter-rater reliability of radiologic measurements was assessed in a subset of 100 patients. All images were independently evaluated by two orthopaedic residents under the supervision of orthopaedic and radiology consultants. Interrater agreement was quantified using intraclass correlation coefficients (ICC) for continuous measurements and Cohen's kappa for binary outcomes. For ordinal variables, weighted kappa was applied to account for the degree of disagreement between categories.

Inter-rater reliability varied across the assessed parameters. Excellent agreement was observed for the hip–knee angles. Good reliability was found for MPTA, JLCA, and the K&L grade, reflecting overall robust reproducibility. Patellar height demonstrated moderate to good agreement, while tibial slope showed only moderate reliability, suggesting greater variability and potential measurement difficulty.

Kellgren-Lawrence Grade

Linear Weighted Kappa = 0.828 (95% CI: 0.747 - 0.900)

Quadratic Weighted Kappa = 0.888 (95% CI: 0.833 - 0.934)

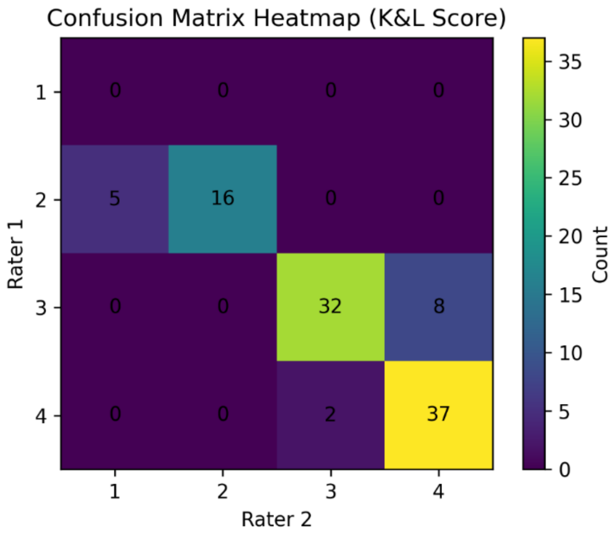

Hip-Knee Angle (HKA) (Varus +, Valgus -)

ICC = 0.994 (95% CI: 0.991 – 0.996)

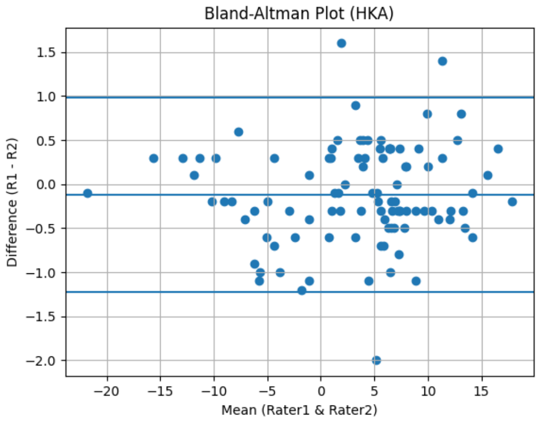

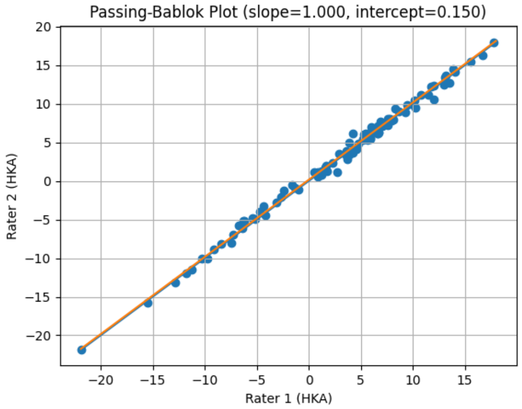

Canton Deschamps Index

ICC = 0.750 (95% CI: 0.465 – 1.000)

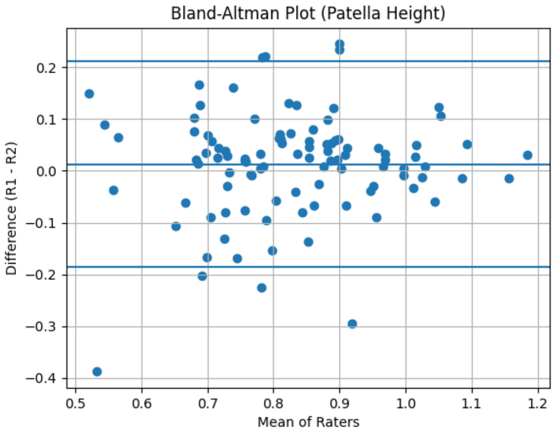

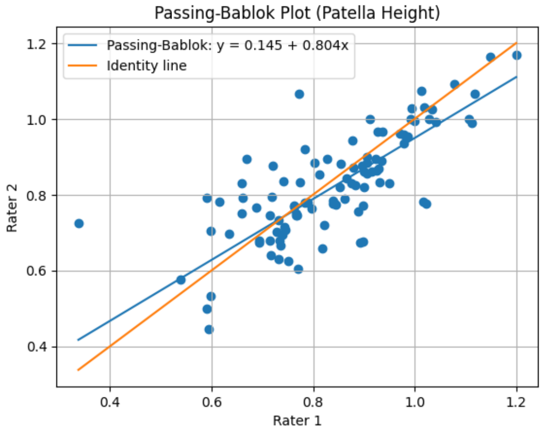

Medial Proximal Tibial Angle (MPTA)

ICC = 0.879 (95% CI: 0.579 – 1.000)

Joint Line Convergence Angle (JLCA)

ICC = 0.818 (95% CI: 0.522 – 1.000)

Tibial Slope

ICC = 0.699 (95% CI: 0.522 – 1.000)
